# Heavy Metal Pollution Characteristics of Surface Sediments in Different Aquatic Ecosystems in Eastern China: A Comprehensive Understanding

**DOI:** 10.1371/journal.pone.0108996

**Published:** 2014-09-30

**Authors:** Wenzhong Tang, Baoqing Shan, Wenqiang Zhang, Hong Zhang, Lishuo Wang, Yuekui Ding

**Affiliations:** 1 State Key Laboratory on Environmental Aquatic Chemistry, Research Center for Eco-Environmental Sciences, Chinese Academy of Sciences, Beijing, China; 2 China University of Mining & Technology (Beijing), Beijing, China; University of Vigo, Spain

## Abstract

Aquatic ecosystems in eastern China are suffering threats from heavy metal pollution because of rapid economic development and urbanization. Heavy metals in surface sediments were determined in five different aquatic ecosystems (river, reservoir, estuary, lake, and wetland ecosystems). The average Cd, Cr, Cu, Ni, Pb, and Zn concentrations were 0.716, 118, 37.3, 32.7, 56.6, and 204 mg/kg, respectively, and the higher concentrations were mainly found in sediment samples from river ecosystems. Cd was the most anthropogenically enriched pollutant, followed by Zn and Pb, indicated by enrichment factors >1.5. According to consensus-based sediment quality guidelines, potential ecological risk indices, and risk assessment codes, all five types of aquatic ecosystems were found to be polluted with heavy metals, and the most polluted ecosystems were mainly rivers. Cd was the most serious pollutant in all five aquatic ecosystems, and it was mainly found in the exchangeable fraction (about 30% of the total Cd concentration, on average). The results indicate that heavy metal contamination, especially of Cd, in aquatic ecosystems in eastern China should be taken into account in the development of management strategies for protecting the aquatic environment.

## Introduction

Heavy metal contamination has been a worldwide environmental problem for many years [1]–[4]. Heavy metals are widespread, persistent, and potentially toxic in aquatic ecosystems [5]–[7]. After being transported into aquatic ecosystems, heavy metals can be absorbed by suspended solids and accumulated in sediments. Sediment acts as a sink for heavy metals but can re-release the heavy metals, acting as a source of pollutants and degrading the quality of the aquatic ecosystems [8], [9]. Therefore, the accumulation of heavy metals in sediment is a cause of growing interest and concern. Environmental problems caused by heavy metal pollution in aquatic ecosystems have recently been extensively studied so that procedures for effectively managing these ecosystems can be developed [10]–[12].

Heavy metal contamination in aquatic ecosystems usually accompanies rapid economic development, in both developed and developing countries [13]. Little attention was paid to environmental problems caused by heavy metal contamination in sediments in China by researchers or the government until 2000 [4], [14]. Economic development, especially industrial development, has recently been continuously and rapidly intensifying in China, particularly in eastern China. Heavy metals discharged into the air and effluents by industrial activities have caused severe pollution in aquatic ecosystems in eastern China [4], [6], [15]. The State Council of China approved the “12^th^ National Five Year Plan for Comprehensive Prevention and Control of Heavy Metal Pollution” in early 2011, with the aim of controlling heavy metal contamination in the environment [16]. Eastern China is the most developed and most densely populated region in China. Rapid economic development and urbanization have caused significant amounts of pollution, including heavy metal pollution, in different aquatic ecosystems (rivers, lakes, and wetlands) in eastern China [6], [13], [15], [16]. It is important to comprehensively understand the characteristics of heavy metal contamination in different types of aquatic ecosystems in this area to provide a reference point for the development of better procedures for controlling and managing heavy metals in the affected ecosystems.

In the study presented here, we evaluated the heavy metal pollution status of five different types of aquatic ecosystems (river, reservoir, estuary, lake, and wetland ecosystems) in eastern China. The specific goals were: 1) to determine heavy metal (Cd, Cr, Cu, Ni, Pb, and Zn) concentrations in surface sediments in twelve study areas; 2) to use enrichment factors (EFs) to determine the importance of lithogenic and anthropogenic sources of the heavy metals; and 3) to assess the risks associated with the presence of the heavy metals in the aquatic ecosystems using consensus-based sediment quality guidelines (SQGs), potential ecological risk (PER) indices, and risk assessment codes (RACs).

## Materials and Methods

### Ethics Statement

No specific permits were required for the field studies described here. The study areas are not privately owned or protected in any way, and the field studies did not involve endangered or protected species.

### Description of the Study Areas

**Figure 1:**
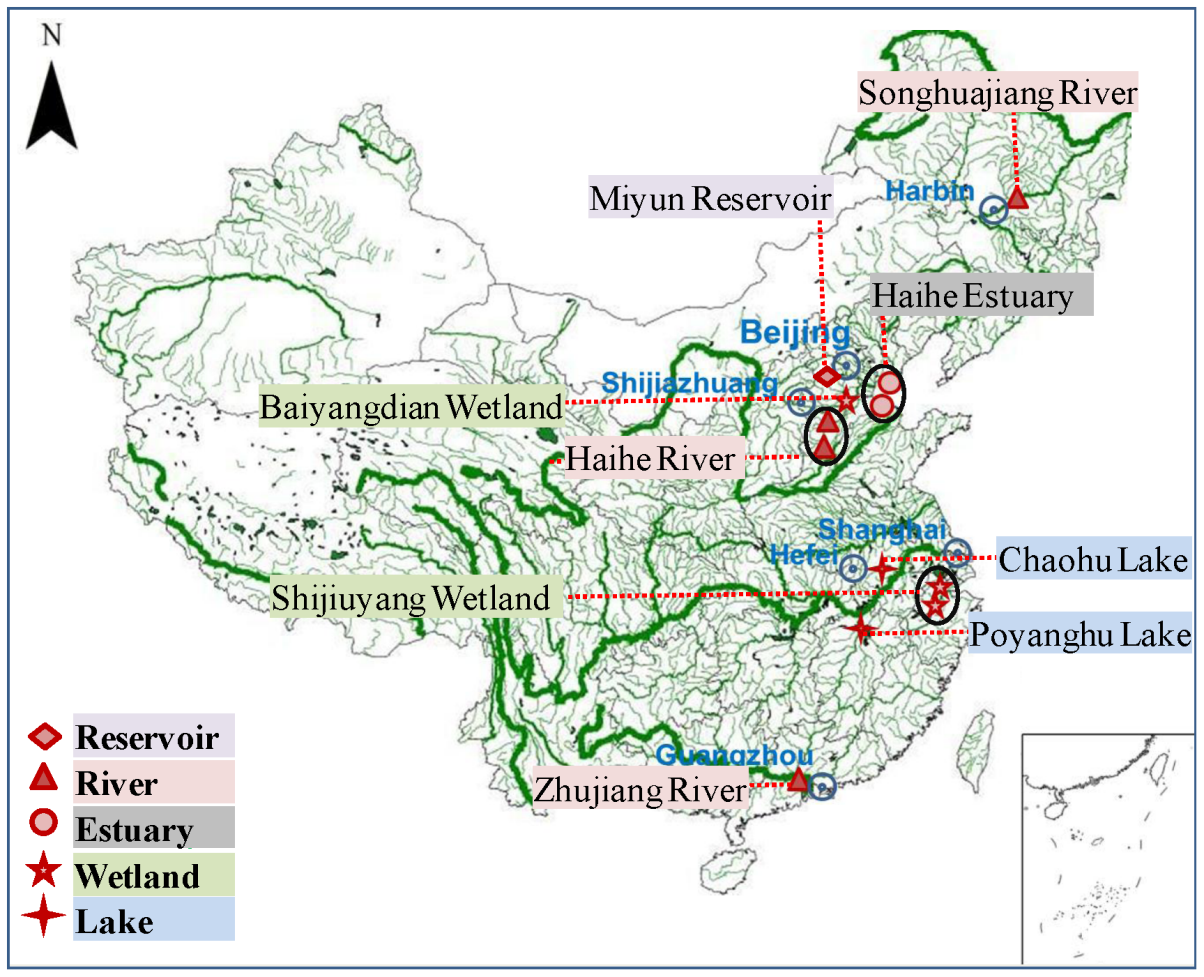
Locations of the five aquatic ecosystem types and the 12 areas that were studied.

Twelve study areas (Fig. 1) in eastern China that have been affected by rapid industrial development and urbanization were selected to represent five typical aquatic ecosystems (reservoirs, rivers, estuaries, wetlands, and lakes). The study areas were in Heilongjiang Province, Heibei Province, Beijing Municipality, Tianjin Municipality, Zhejiang Province, Anhui Province, Jiangxi Province, and Guangdong Province. The study areas were in the region between 113° 15′ 31.08″ E and 126° 30′ 52.43″ E and between 23° 50′ 26.85″ N and 45° 46′ 18.01″ N.

The river ecosystems were the Songhuajiang River (SR), two typical rivers in the Haihe River basin (HR1 and HR2), and the Zhujiang River (ZR). The reservoir ecosystem was the Miyun Reservoir (MRe). Estuarine ecosystems were represented by the estuaries of rivers HR1 (estuary HE1) and HR2 (estuary HE2). The lake ecosystems were Chaohu Lake (CL) and Poyanghu Lake (PL), and the wetland ecosystems were the Baiyangdian Wetland (BW) and two areas in the Shijiuyang Wetland (SW1 and SW2).

### Sample Collection and Analysis

Surface sediment samples were collected from the twelve study areas (Fig. 1) using a Peterson grab sampler between August and October 2013. Five sampling stations were established in each study area, and three parallel samples were taken from each station (Fig. S1 in File S1). The samples (n = 180) were air-dried in the laboratory then dried in an oven at 40°C. The dried samples were ground and passed through a 100 mesh sieve before they were analyzed.

For the total heavy metal analysis, each sediment sample (0.100 g) was digested in a 5∶1 mixture of hydrofluoric acid and perchloric acid [17] in a microwave digestion system (MARS XPress; CEM, Matthews, NC, USA) using the digestion conditions shown in Table S1 in File S1. The geochemical fractionation of the heavy metals in a sample was determined using the BCR three-step sequential extraction procedure [18], to determine the exchangeable (sediment solution, carbonates, and exchangeable metals), reducible (Fe/Mn oxides), oxidizable (organic matter and sulfides), and residual (remaining non-silicate bound) fractions of the heavy metals. All of the above solutions were stored at 4°C until instrumental analysis was performed. The Cd, Cr, Cu, Fe, Ni, Pb, and Zn concentrations in the extracts were determined using an inductively coupled plasma-mass spectrometry (ICP-MS) instrument (7500a; Agilent Technologies, Santa Clara, CA, USA), which gave detection limits of 0.015–0.120 µg/L, and an inductively coupled plasma optical emission spectrometry (ICP-OES) instrument (Optima 2000DV; Perkin Elmer, Waltham, MA, USA), which gave detection limits of 0.001–0.030 mg/L. The quality control procedures used in the laboratory were analyzing a sediment reference material (GBW07302a; National Institute of Metrology, Beijing, China) and analyzing samples in triplicate. The recoveries of the heavy metals varied but were all within the range 90–105%. The analytical method gave a good level of precision, the relative standard deviation being less than 5%. Each result that is presented is the average of the measurements made at the five sampling stations in each study area.

### Enrichment Factors

The EFs for the heavy metals in the surface sediment samples were calculated using eq. 1, which was published by Zhang et al. [19], to provide information on the sources of the metal contaminants and temporal variations in the contributions of the different sources.
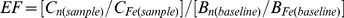
(1)where *C_n_* is the metal concentration in a sediment, *B_n_* is the background concentration of the metal, *C_Fe_* is the Fe concentration in the sediment, and *B_Fe_* is the background Fe concentration. The background metal concentrations in soils from each of the twelve study areas were used as the baseline concentrations [20].

### Risk Assessment

The ecological risks posed by heavy metals in surface sediments were assessed using three methods, consensus-based SQGs [7], PER indices [15], [21], and RACs [22]. Details of the assessment methods are provided in Table S2 in File S1.

## Results and Discussion

### Heavy Metal Concentrations in Surface Sediments

**Figure 2:**
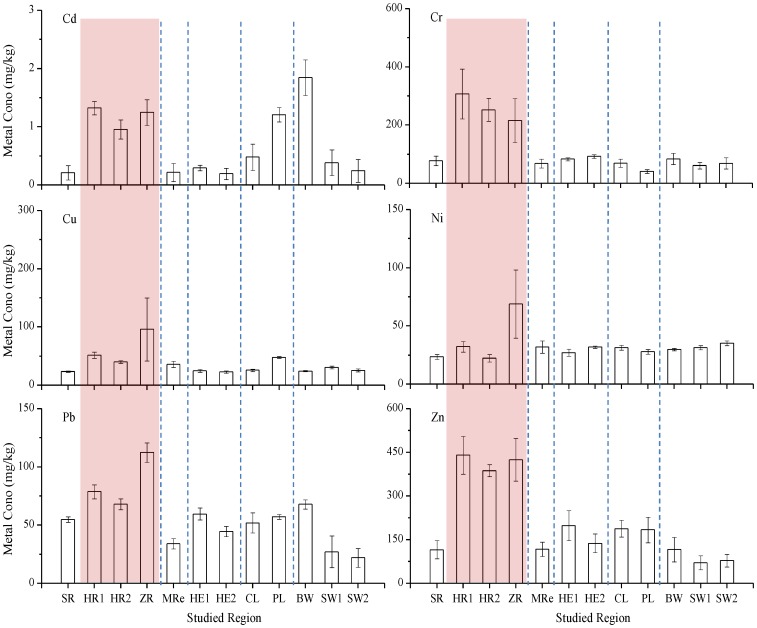
Heavy metal concentrations in the surface sediment samples from each of the study sites.

The heavy metal concentrations found in the surface sediments from the five types of aquatic ecosystems are shown in Fig. 2. The average Cd, Cr, Cu, Ni, Pb, and Zn concentrations in the twelve study areas were 0.716, 118, 37.3, 32.7, 56.6, and 204 mg/kg, respectively. The highest average Cd concentrations (>1.00 mg/kg) were found in surface sediments from the HR1, ZR, PL, and BW sites. The highest average Cr concentrations were found in surface sediments from the HR1, HR2, and ZR sites (307, 252, and 215 mg/kg, respectively). The highest average Cu, Ni, and Pb concentrations (96.1, 68.8, and 112 mg/kg, respectively) were found in surface sediments from the ZR site. The highest average Zn concentrations were found in surface sediments from the HR1, HR2, and ZR sites (440, 387, and 424 mg/kg, respectively). The highest total heavy metal concentrations (i.e., the sum of the Cd, Cr, Cu, Ni, Pb, and Zn concentrations) in surface sediments were mainly found in samples from river ecosystems (Fig. 1 and Fig. 2), especially samples from the HR1 and ZR sites, which contained total heavy metal concentrations of 911 and 918 mg/kg, respectively. The high heavy metal concentrations in the surface sediments from the rivers implied that the rivers in the study area have suffered substantial inputs of heavy metals from anthropogenic sources [23].

The relationships between the concentrations of different heavy metals elements can provide information on the sources of the heavy metal [24]. Therefore, the Spearman correlation coefficients for the relationships between the heavy metal concentrations were determined (Table S3 in File S1). Positive correlations (p≤0.01) were found between the Cd and Pb concentrations and between the Pb and Zn concentrations. Significant positive correlations (p≤0.05) were also found between the Cd and Cu concentrations, the Cd and Zn concentrations, the Cr and Pb concentrations, and the Cr and Zn concentrations.

### Heavy Metal Sources to the Surface Sediments

**Figure 3:**
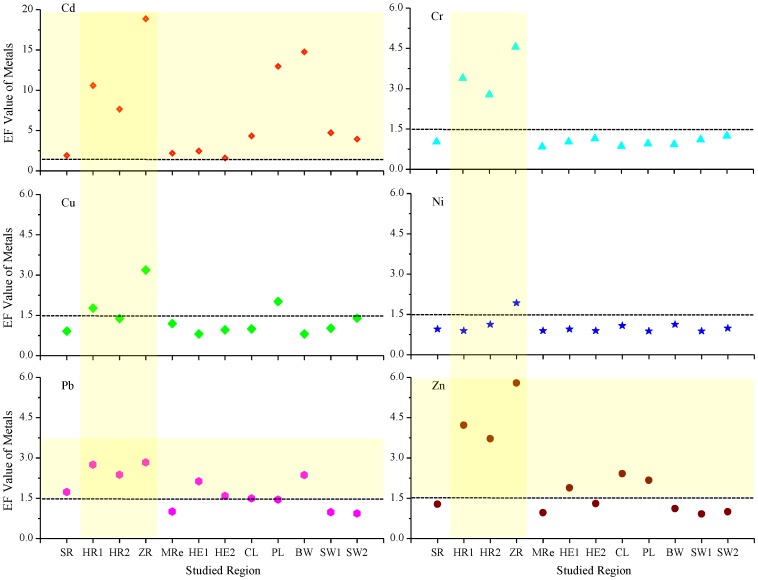
Heavy metal enrichment factors (EFs) in the sediment samples from the study sites.

The EF is a normalization technique that is widely used to identify the proportion of a metal derived from natural environmental sources and the proportion of the metal derived from anthropogenic activities [16], [25]. The EF for each metal was calculated, and is shown in Fig. 3, to determine the anthropogenic influences on the heavy metal concentrations in surface sediments in the five types of aquatic ecosystems that were studied in the study areas. The mean EF was highest for Cd (7.16), indicating that anthropogenic sources had had a stronger impact on Cd concentrations than the concentrations of the other heavy metals in the sediments in the study areas. The remaining EFs decreased in the order Zn (2.23), Pb (1.81), Cr (1.65), Cu (1.37), and Ni (1.05). The EFs were highest for the surface sediments from the river sites, and the average EFs were 3.93, 3.17, and 6.18 for the samples from the HR1, HR2, and ZR sites, respectively. The spatial distribution of the EFs for each metal was similar to the spatial distribution of the concentrations of that metal.

An EF of approximately 1 suggests that the metal may originate entirely from natural sources, such as crustal material and natural weathering processes. An EF slightly higher than 1 may not necessarily be caused by the addition of the metal from anthropogenic activities, but may be caused by natural differences in the metal concentrations in the sediment and the reference soil used for the EF calculation. Therefore, an EF of between 0.5 and 1.5 suggests that the metal may be entirely derived from crustal materials and natural weathering processes. However, an EF higher than 1.5 suggests that a significant proportion of the metal originates from anthropogenic processes [16], [25], [26].

The Cd, Pb, and Zn concentrations in the sediments from the aquatic ecosystems in the study areas positively correlated with each other (Table S3 in File S1) and these were found to be the most anthropogenically enriched of the heavy metals that were analyzed at most of the study sites (EF>1.5) (Fig. 3). The potential anthropogenic sources of these metals include industrial processes, agriculture, and mining [16], [27], [28]. The mean EF for Cr in the sediments that were analyzed was slightly higher than 1.5, and this was mainly caused by high EFs at the HR1, HR2, and ZR sites (3.39, 278, and 4.55, respectively). This indicates that Cr was enriched in the sediments at the HR1, HR2, and ZR sites because of anthropogenic emissions. The mean EFs for Cu and Ni were less than 1.5, indicating that these two metals were mainly derived from natural sources, such as the underlying geological material. The EFs that were obtained may be useful indicators of the role of anthropogenic processes in the distributions of the metals in the study area. The mean EFs for Cd in the sediment samples from the five types of aquatic ecosystems were all greater than 1.5, and the Cd EFs were particularly high at the ZR (18.9), BW (14.8), and PL (13.0) sites (Fig. 3). These results indicate that Cd pollution has become a major environmental problem in aquatic ecosystems in eastern China, and this conclusion is consistent with the conclusions of other studies [4], [6], [16], [27].

### Risks Posed by Heavy Metals in Surface Sediments

**Figure 4:**
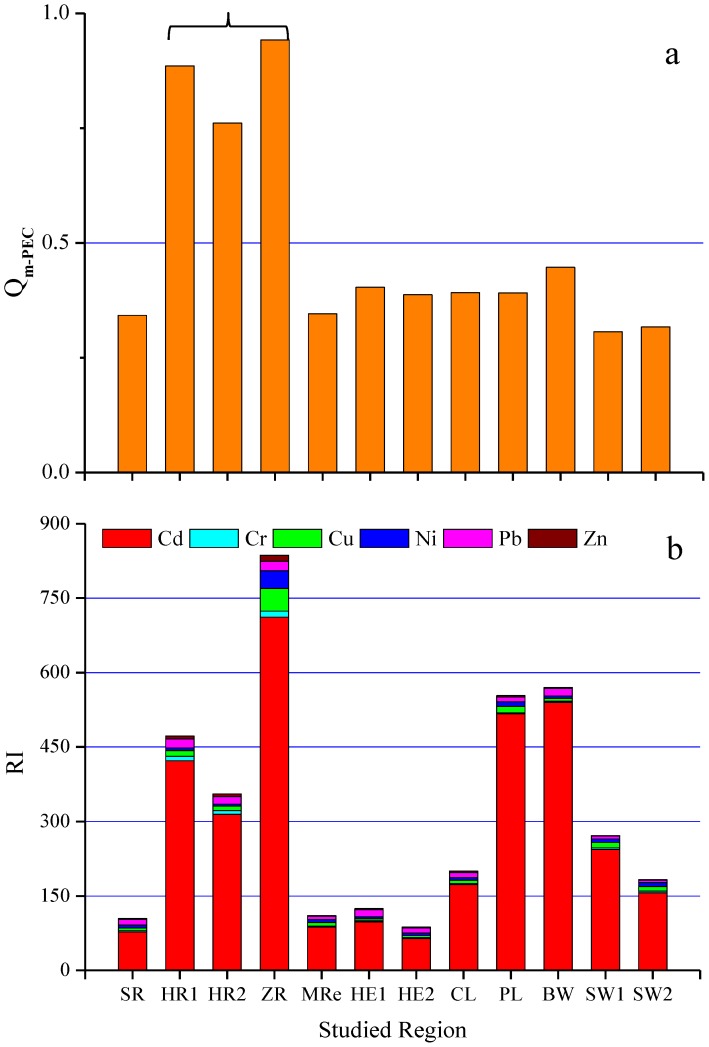
Heavy metal risk assessment indices for the sediment samples from each study site. (a) *Q_m-PEC_* and (b) comprehensive PER index (*RI*).

The ecological risks posed to the five types of aquatic ecosystems that were studied by the heavy metals that were measured in surface sediments were assessed using consensus-based SQGs, PER indices, and RACs. The mean probable effect concentration quotients (*Q_m-PEC_s*) for the sediments from sites HR1, HR2, and ZR were higher than 0.5 (0.885, 0.761, and 0.942, respectively), which indicates that these sediments were toxic and were seriously contaminated with heavy metals (Fig. 4a). The PER index for an individual element (*E^i^_r_*) describes the sensitivity of a biological community to a given substance and can be used to illustrate the risk posed to that community by contaminants [4], [28]. For the aquatic ecosystems in eastern China that we studied, the *E^i^_r_* values for the heavy metals decreased in the order Cd>Cu>Pb>Ni>Cr>Zn. All of the metals that we studied apart from Cd had low PER indices. The *E^i^_r_* values for Cd ranged from 64.2 (at HE2) to 711 (at ZR), and the average was 283, indicating that sediments in aquatic ecosystems in eastern China are highly contaminated with Cd, which is consistent with the conclusions we drew from the Cd EFs.

The comprehensive PER index (*RI*) distribution can be useful for identifying sites that need more attention than others because of risks associated with pollutants [16]. In this study, the mean RI values for the surface sediments were highest at the ZR, BW, and PL sites (836, 570, and 554, respectively), followed by the HR1 and HR2 sites (472 and 356, respectively), and the overall average RI was 322. According to the PER index criteria (Table S2 in File S1), the comprehensive PER for the surface sediments at the ZR site showed that this site suffers from very high-grade risks. The BW, PL, HR1, and HR2 sites all had high-grade risks, and the CL, SW1, and SW2 sites had moderate-grade risks. The sediments in the river ecosystems had higher PER indices than did the sediments from the other ecosystems, mainly resulting from Cd pollution, and this conclusion was similar to the conclusions we drew from the distributions of the heavy metal concentrations and EFs. Heavy metal pollution was found to be most severe in the river ecosystems, and this is probably because rivers, being open systems, are more vulnerable to contamination caused by anthropogenic processes. Pollutants are often discharged directly into rivers, and they can be quickly deposited in river sediments. The pollutants that are not deposited in the sediments will be transported to other ecosystems in the flowing water [6].

**Figure 5:**
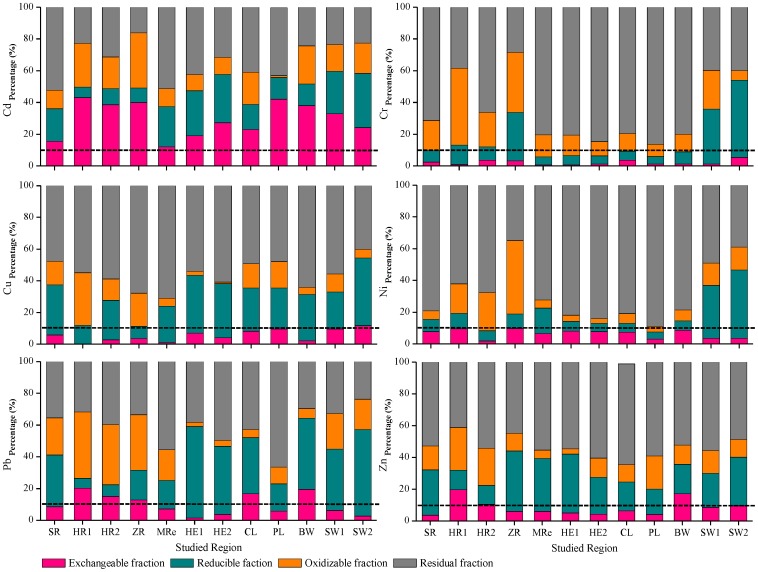
Proportions of the heavy metal fractions in sediments from the five aquatic ecosystem types.

The Cd, Cr, Cu, Ni, Pb, and Zn speciation in the sediment samples was investigated (Fig. 5) to further evaluate the heavy metal contamination in the sediment samples. Cr, Cu, Ni, and Zn were predominantly found in the residual fraction in the studied sediments, and this fraction contained an average of more than 50% of the total metal content. In contrast, about 30% of the total Cd content and about 10% of the total Pb content were found in the exchangeable fraction of each sample. A high proportion of a metal in the exchangeable fraction indicates anthropogenic pollution [29], [30]. According to the RAC classification (Table S2 in File S1), the risks posed by Cd in the surface sediments were high at the HR1, HR2, ZR, PL, BW, and SW1 sites and medium at the other sites. The risks posed by Pb in the surface sediments were found to be medium at the HR1, HR2, ZR, CL, and BW sites, and the risks posed by Zn were also found to be medium at the HR1, HR2, and BW sites. These results corroborate the EF and PER index results. Contaminants in the sediment may move into the foodchain, particularly if the contaminants are in bioavailable forms. Cd has a high transfer rate in the environment, so it can accumulate in relatively large amounts in plants without any apparent effects on the plants. Cd can, therefore, be found in most human foodstuffs, and this can cause health problems in humans [16], [31], [32]. Therefore, heavy metal contaminants, especially Cd, in aquatic ecosystems (particularly river ecosystems) in eastern China should be carefully monitored and controlled in the future [6], [33].

## Conclusions

We studied heavy metal pollutants in surface sediments from different aquatic ecosystems in eastern China. The heavy metal concentrations were usually higher in surface sediments in river ecosystems than in other ecosystems. The EFs showed that Cd, Zn, and Pb were enriched in the surface sediments more because of anthropogenic sources than were Cr, Cu, and Ni, which were predominantly derived from natural sources in most of the study areas. Using consensus-based SQGs, PER indices, and RACs, we found that all five types of aquatic ecosystems, especially river ecosystems, in the study areas have been polluted with heavy metals. The most serious pollutant in the study areas is Cd, which was mainly found in the exchangeable fraction of the sediment samples. This information could be useful for the development of effective management strategies for controlling heavy metal pollution in aquatic ecosystems in eastern China.

## Supporting Information

File S1 **Table S1.** Microwave digestion conditions used for the sediment samples. **Table S2.** The evaluation criteria for different risk assessment methods. **Table S3.** Correlation coefficients for the heavy metals in the surface sediments from different types of aquatic ecosystems. **Figure S1.** Map showing the layouts used at the sampling stations in each type of aquatic ecosystem.
(DOCX)
