# Supplementary material for: Heavy Metal Pollution Characteristics of Surface Sediments in Different Aquatic Ecosystems in Eastern China: A Comprehensive Understanding
Source: PLoS One. 2014 Sep 30;9(9):e108996. doi: 10.1371/journal.pone.0108996 (PMC4182660; doi:10.1371/journal.pone.0108996)
Supplement: File S1 — Table S1. Microwave digestion conditions used for the sediment samples. Table S2. The evaluation criteria for different risk assessment methods. Table S3. Correlation coefficients for the heavy metals in the surface sediments from different types of aquatic ecosystems. Figure S1. Map showing the layouts used at the sampling stations in each type of aquatic ecosystem. (DOCX) [file pone.0108996.s001.docx]

**Supporting Information**

**Heavy Metal Pollution Characteristics of Surface Sediments in Different Aquatic** **Ecosystems in Eastern China: A Comprehensive Understanding**

Wenzhong Tang ^a,^*, Baoqing Shan ^a,^*, Wenqiang Zhang ^a^, Hong Zhang ^a^, Lishuo Wang ^a, b^, Yuekui Ding ^a^

^a^ State Key Laboratory on Environmental Aquatic Chemistry, Research Center for Eco-Environmental Sciences, Chinese Academy of Sciences, Beijing, China

^b^ China University of Mining & Technology (Beijing), Beijing, China

* E-mail: [wztang@rcees.ac.cn](mailto:wztang@rcees.ac.cn) (WZT), [bqshan@rcees.ac.cn](mailto:bqshan@rcees.ac.cn) (BQS)

**Contents:**

1. **Microwave digestion conditions used for the sediment samples (Table S1) 2**
2. **Risk assessment methods (containing Table S2) 3**
3. **Correlation coefficients for the heavy metals in the surface sediments from different types of aquatic ecosystems (Table 3) 5**
4. **Map showing the layouts used at the sampling stations** **in each type of aquatic ecosystem (Fig. S1) 7**
5. **References 8**

**Table S1.** Microwave digestion conditions used for the sediment samples.

| **Stage** | **Power** | | **Ramp (min)** | **Temperature (°C)** | **Hold (min)** |
| --- | --- | --- | --- | --- | --- |
|  | **Max (W)** | **Percentage** |  |  |  |
| 1 | 1600 | 100% | 6 | 120 | 2 |
| 2 | 1600 | 100% | 5 | 150 | 5 |
| 3 | 1600 | 100% | 5 | 180 | 20 |

# Risk Assessment Methods

## Consensus-based Sediment Quality Guidelines

A consensus-based sediment quality guideline (SQG) method was developed by Macdonald et al. [1]. There are two consensus-based values, the threshold effect concentration (TEC), below which adverse effects are not expected to occur, and the probable effect concentration (PEC), above which adverse effects are expected to occur more often than not [2]. The mean PEC quotient (*Q_m-PEC_*) was introduced to predict the toxicity of a sediment sample, and this can be calculated using eq. 1,

$Q_{m-PEC} = \overline{\sum C_{n}/PEC_{n}}$ (1)

where *C_n_* is the measured concentration of a heavy metal and *PEC_n_* is the corresponding PEC. The PEC benchmark values for Cd, Cr, Cu, Ni, Pb, and Zn in sediment samples are 4.98, 111, 149, 48.6, 128, and 459 mg/kg, respectively. The evaluation criteria for *Q_m-PEC_* are listed in Table S2.

## Potential Ecological Risk

The potential ecological risk (PER) index was used to assess the degree of heavy metal contamination in the sediments. The equations for calculating the PER index were proposed by Guo et al. [3] and are shown in eqs. 2 and 3,

$E_{r}^{i}=T_{r}^{i}\times C_{f}^{i}=T_{r}^{i}\times(C_{s}^{i}/C_{n}^{i})$ (2)

 (3)

where *C^i^_s_* is the element concentration in the sample, *C^i^_n_* is the reference value for the element, *C^i^_f_* is the single element pollution factor, *E^i^_r_* is the PER index for an individual element, and *T^i^_r_* is the biological toxicity factor of an individual element, defined as Cd = 30, Cr = 2, Cu = Ni = Pb = 5, and Zn = 1 [3,4]. *RI* is the comprehensive PER index, which is the sum of the *E^i^_r_* values. Table S2 shows the standard factors for different levels of risk.

## Risk Assessment Codes

The risk assessment codes (RACs) were used in this study to assess the heavy metal content of the sediments from a regulatory perspective. The RAC allows the potential risk of a heavy metal being released into solution to be assessed by calculating the percentage of the metal in the exchangeable fraction in the sediment [5]. The evaluation criteria for the RACs are listed in Table S2.

**Table S2.** The evaluation criteria for different risk assessment methods.

| **Consensus-based SQGs** | | | |
| --- | --- | --- | --- |
| ***Q_m-PEC_*** | **Sediment quality** |  |  |
| < 0.5 | Not toxic |  |  |
| > 0.5 | Toxic |  |  |
| **Potential Ecological Risk (PER) Index** | | | |
| **E_i_^r^** | **PER of individual elements** | **RI** | **Comprehensive PER** |
| <40 | Low | <150 | Low |
| 40–80 | Moderate | 150-300 | Moderate |
| 80–160 | Considerable | 300-600 | High |
| 160–320 | High | ≥600 | Very high |
| ≥320 | Very high |  |  |
| **Risk Assessment Codes (RACs)** | | | |
| **Proportion of metals in the exchangeable fraction** | | **Potential release risk** | |
| <1% | | no risk | |
| 1–10% | | low risk | |
| 11–30% | | medium risk | |
| 31–50% | | high risk | |
| ≥75% | | very high risk | |

**Table S3.** Correlation coefficients for the heavy metals in the surface sediments from different types of aquatic ecosystems.

|  |  | **Cd** | | | | | **Cr** | | | | | **Cu** | | | | | **Ni** | | | | | **Pb** | | | | | **Zn** | | | | |
| --- | --- | --- | --- | --- | --- | --- | --- | --- | --- | --- | --- | --- | --- | --- | --- | --- | --- | --- | --- | --- | --- | --- | --- | --- | --- | --- | --- | --- | --- | --- | --- |
|  |  | Tot | Exc | Red | Oxi | Res | Tot | Exc | Red | Oxi | Res | Tot | Exc | Red | Oxi | Res | Tot | Exc | Red | Oxi | Res | Tot | Exc | Red | Oxi | Res | Tot | Exc | Red | Oxi | Res |
| **Cd** | Tot | 1 |  |  |  |  |  |  |  |  |  |  |  |  |  |  |  |  |  |  |  |  |  |  |  |  |  |  |  |  |  |
|  | Exc | .951^a^ | 1 |  |  |  |  |  |  |  |  |  |  |  |  |  |  |  |  |  |  |  |  |  |  |  |  |  |  |  |  |
|  | Red | .832^a^ | .895^a^ | 1 |  |  |  |  |  |  |  |  |  |  |  |  |  |  |  |  |  |  |  |  |  |  |  |  |  |  |  |
|  | Oxi | .734^a^ | .636^b^ | .510 | 1 |  |  |  |  |  |  |  |  |  |  |  |  |  |  |  |  |  |  |  |  |  |  |  |  |  |  |
|  | Res | .846^a^ | .769^a^ | .629^b^ | .385 | 1 |  |  |  |  |  |  |  |  |  |  |  |  |  |  |  |  |  |  |  |  |  |  |  |  |  |
| **Cr** | Tot | .322 | .301 | .056 | .559 | .252 | 1 |  |  |  |  |  |  |  |  |  |  |  |  |  |  |  |  |  |  |  |  |  |  |  |  |
|  | Exc | .238 | .266 | .098 | .545 | -.007 | .650^b^ | 1 |  |  |  |  |  |  |  |  |  |  |  |  |  |  |  |  |  |  |  |  |  |  |  |
|  | Red | .385 | .406 | .336 | .699^b^ | -.028 | .594^b^ | .755^a^ | 1 |  |  |  |  |  |  |  |  |  |  |  |  |  |  |  |  |  |  |  |  |  |  |
|  | Oxi | .280 | .189 | .070 | .524 | .140 | .629^b^ | .392 | .664^b^ | 1 |  |  |  |  |  |  |  |  |  |  |  |  |  |  |  |  |  |  |  |  |  |
|  | Res | .301 | .280 | .035 | .399 | .399 | .909^a^ | .413 | .252 | .427 | 1 |  |  |  |  |  |  |  |  |  |  |  |  |  |  |  |  |  |  |  |  |
| **Cu** | Tot | .594^b^ | .497 | .462 | .343 | .524 | .105 | .231 | .322 | .413 | .000 | 1 |  |  |  |  |  |  |  |  |  |  |  |  |  |  |  |  |  |  |  |
|  | Exc | .028 | .049 | .196 | -.140 | -.042 | -.462 | .112 | -.126 | -.406 | -.517 | .147 | 1 |  |  |  |  |  |  |  |  |  |  |  |  |  |  |  |  |  |  |
|  | Red | -.308 | -.252 | .035 | -.538 | -.119 | -.329 | -.133 | -.238 | -.413 | -.238 | .049 | .259 | 1 |  |  |  |  |  |  |  |  |  |  |  |  |  |  |  |  |  |
|  | Oxi | .497 | .462 | .406 | .259 | .434 | .119 | .336 | .448 | .531 | -.070 | .867^a^ | .119 | -.014 | 1 |  |  |  |  |  |  |  |  |  |  |  |  |  |  |  |  |
|  | Res | .531 | .441 | .420 | .357 | .538 | .336 | .042 | .252 | .580^b^ | .301 | .797^a^ | -.308 | -.098 | .678^b^ | 1 |  |  |  |  |  |  |  |  |  |  |  |  |  |  |  |
| **Ni** | Tot | .063 | .056 | .049 | .238 | -.266 | .056 | .182 | .392 | .070 | -.203 | .329 | -.021 | -.182 | .196 | .294 | 1 |  |  |  |  |  |  |  |  |  |  |  |  |  |  |
|  | Exc | .294 | .210 | .014 | .469 | .147 | .545 | .154 | .280 | .273 | .420 | .007 | -.257 | -.622^b^ | -.091 | .245 | .531 | 1 |  |  |  |  |  |  |  |  |  |  |  |  |  |
|  | Red | -.091 | -.119 | -.063 | .301 | -.503 | -.133 | .182 | .531 | .245 | -.476 | .112 | -.049 | -.287 | .196 | .021 | .727^a^ | .224 | 1 |  |  |  |  |  |  |  |  |  |  |  |  |
|  | Oxi | .566 | .497 | .406 | .825^a^ | .182 | .448 | .706^b^ | .867^a^ | .622^b^ | .161 | .608^b^ | -.049 | -.294 | .608^b^ | .448 | .427 | .210 | .538 | 1 |  |  |  |  |  |  |  |  |  |  |  |
|  | Res | .070 | .056 | .007 | -.175 | .273 | -.007 | -.308 | -.538 | -.364 | .168 | -.070 | .175 | -.175 | -.245 | .133 | .112 | .497 | -.406 | -.476 | 1 |  |  |  |  |  |  |  |  |  |  |
| **Pb** | Tot | .706^a^ | .615^b^ | .441 | .545 | .762^a^ | .727^b^ | .378 | .399 | .573 | .699^b^ | .462 | -.161 | -.210 | .462 | .573 | -.140 | .434 | -.350 | .399 | .147 | 1 |  |  |  |  |  |  |  |  |  |
|  | Exc | .748^a^ | .650^a^ | .385 | .671^b^ | .727^a^ | .573 | .420 | .378 | .545 | .510 | .517 | -.203 | -.559 | .580^b^ | .601^b^ | .028 | .448 | -.063 | .559 | .154 | .790^a^ | 1 |  |  |  |  |  |  |  |  |
|  | Red | -.070 | -.091 | .035 | .091 | -.140 | .063 | -.105 | -.063 | -.217 | .049 | -.538 | .273 | -.091 | -.580^b^ | -.406 | -.042 | 0455 | -.070 | -.287 | .406 | .070 | -.196 | 1 |  |  |  |  |  |  |  |
|  | Oxi | .336 | .366 | .175 | .350 | .266 | .315 | .448 | .559 | .699^b^ | .084 | .699^b^ | -.182 | -.133 | .895^a^ | .664^b^ | .224 | .021 | .343 | .685^b^ | -.357 | .448 | .636^b^ | -.537 | 1 |  |  |  |  |  |  |
|  | Res | .545 | .510 | .406 | .126 | .748^a^ | .413 | .203 | .014 | .210 | .503 | .559 | .196 | .126 | .462 | .531 | -.196 | .154 | -.636^b^ | .077 | .406 | .797^a^ | .531 | -.042 | .252 | 1 |  |  |  |  |  |
| **Zn** | Tot | .676^b^ | .614^b^ | .147 | .336 | .580^b^ | .671^b^ | .371 | .217 | .441 | .692^b^ | .573 | -.063 | -.091 | .378 | .573 | .042 | .406 | -.406 | .301 | .280 | .804^a^ | .573 | -.098 | .308 | .846^a^ | 1 |  |  |  |  |
|  | Exc | .804^a^ | .699^b^ | .510 | .797^a^ | .643^b^ | .629^b^ | .545 | .538 | .413 | .580^b^ | .587^b^ | -.091 | -.210 | .378 | .483 | .133 | .343 | -.091 | .706^b^ | -.084 | .734^a^ | .664^b^ | -.126 | .336 | .545 | .734^a^ | 1 |  |  |  |
|  | Red | .126 | -.070 | -.210 | .210 | .245 | .580^b^ | .308 | .224 | .545 | .517 | .441 | -.098 | -.014 | .294 | .448 | .077 | .385 | -.168 | .266 | .098 | .636^b^ | .364 | .007 | .357 | .580^b^ | .846^a^ | .503 | 1 |  |  |
|  | Oxi | .552 | .594^b^ | .329 | .336 | .580^b^ | .559 | .615^b^ | .364 | .399 | .503 | .531 | .077 | -.238 | .650^b^ | .441 | -.063 | .147 | -.294 | .420 | .119 | .692^b^ | .762^a^ | -.350 | .566 | .727^a^ | .657^b^ | .552 | .343 | 1 |  |
|  | Res | .420 | .322 | .140 | .259 | .580^b^ | .573 | .413 | .126 | .413 | .622^b^ | .608^b^ | .070 | -.028 | .469 | .580^b^ | -.105 | .196 | -.490 | .273 | .287 | .748^a^ | .601^b^ | -.203 | .392 | .881^a^ | .937^a^ | .636^b^ | .769^a^ | .755^a^ | 1 |
| ^a^ Correlation is significant at the *p =* 0.01 level (2-tailed).  ^b^ Correlation is significant at the *p =* 0.05 level (2-tailed). | | | | | | | | | | | | | | | | | | | | | | | | | | | | | | | |

Tot, total content; Exc, exchangeable fraction; Red, reducible faction; Oxi, oxidizable faction; Res, residual fraction.

**Figure S1. Map showing the layouts used at the sampling stations in each type of aquatic ecosystem.**

**References:**

1. Macdonald DD, Ingersoll CG, Berger TA (2000) Development and evaluation of consensus-based sediment quality guidelines for freshwater ecosystems. Arch Environ Con Tox 39: 20-31.

2. Niu H, Deng W, Wu Q, Chen X (2009) Potential toxic risk of heavy metals from sediment of the Pearl River. J Environ Sci 21: 1053-1058.

3. Guo WH, Liu XB, Liu ZG, Li GF (2010) Pollution and potential ecological risk evaluation of heavy metals in the sediments around Dongjiang Harbor, Tianjin. Procedia Environ Sci 2: 729-736.

4. Hakanson L (1980) An ecological risk index for aquatic pollution control: A sedimentological approach. Water Res 14: 975-1001.

5. Singh KP, Mohan D, Singh VK, Malik A (2005) Studies on distribution and fractionation of heavy metals in Gomti river sediments-a tributary of the Ganges, India. J Hydrol 312: 14-27.
